# The Price He Paid

**Published:** 1913-06

**Authors:** Ella Wheeler Wilcox


### The Price He Paid.

BY ELLA WHEELER WILCOX.

I said I would have my fling,
  And do what a young man may:
And I didn't believe a thing
  That the parsons have to say.

I didn't believe in a God
  That gives us blood like fire,
Then flings us into hell because
  We answer the call of desire.

And I said: "Religion is rot,
  And the laws of the world are nil;
For the bad man is he who is caught
  And can not foot his bill.
And there is no place called hell;
  And heaven is only a truth,
When a man has his way with a maid,
  In the fresh keen hour of youth.

"And money can buy us grace,
  If it rings on the plate of the church;
And money can neatly erase,
  Each sign of a sinful smirch."
For I saw men everywhere,
  Hotfooting the road of vice;
And women and preachers smiled on them
  As long as they paid the price.

So I had my joy of life:
  I went the pace of the town;
And then I took me a wife,
  And started to settle down.
I had gold enough and to spare
  For all of the simple joys
That belong with a house and a home
  And a brood of girls and boys.

I married a girl with health
  And virtue and spotless fame.
I gave in exchange my wealth
  And proud old family name.
And I gave her the love of a heart
  Grown sated and sick of sin!
My deal with the devil was all cleaned up,
  And the last bill handed in.

She was going to bring me a child,
  And when in labor she cried,
With love and fear I was wild—
  But now I wish she had died.

For the son she bore me was blind
  And crippled and weak and sore
And his mother was left a wreck.
  It was so she settled my score.

I said I must have my fling,
  And they knew the path I would go,
Yet no one told me a thing
  Of what I needed to know.
Folks talk too much of a soul
  From heavenly joys debarred—
And not enough of the babies unborn
  By the sins of their fathers scarred.

—*Cosmopolitan.*

